# First line treatment of acute and chronic ATLL with zidovudine (AZT) and interferon alpha (IFN-α): haematological and molecular responses

**DOI:** 10.1186/1742-4690-8-S1-A53

**Published:** 2011-06-06

**Authors:** Andrew Hodson, Maria A Demontis, Nicolas Gillet, Lucy Cook, Charles R M Bangham, Paul Fields, Graham P Taylor

**Affiliations:** 1 Section of Infectious Diseases, Wright-Fleming Institute, Imperial College London, London, UK; 2 Department of Immunology, Wright-Fleming Institute, Imperial College London, London, UK; 3 Department of Haematology, Guy’s and St Thomas’ NHS Foundation Trust, London, UK

## Introduction

Recent data suggest an important role of zidovudine (ZDV) and interferon-α (IFN-α) in improving response rates and survival in acute ATLL. Treatment of chronic ATLL with ZDV/IFN-α alone has recently been associated with 100% survival beyond five years.

## Methods

Retrospective analysis of patients with acute and chronic ATLL treated with ZDV/IFN-α first line. Response was assessed one month from the start of treatment using total lymphocyte and CD4 count, HTLV-1 proviral load (PVL) and clonal analysis (in house method).

## Results

Acute ATLL: response rate 33% (1 CR, 2 PR). Median overall survival (OS) 3 months (range 3-8).

Chronic ATLL: response rate 100% (4 CR, 1 PR). Median OS 20 months (range 9-73). In chronic ATLL these prolonged responses were observed despite lower dose therapy. Two patients, showed 10-fold reductions in PVL which occurred more than 1 year after haematological CR. All patients remain in remission at time of analysis. Clonality studies demonstrated a dominant clone at base line with emergence of a polyclonal pattern after viral load reduction.

## Discussion

The complete response in one patient with acute ATLL supports the recent observation that ZDV/IFN-α is effective as first line treatment in some patients.

The significant reduction in PVL and late emergence of a polyclonal integration pattern suggest benefit from prolonged ZDV/IFN-a therapy in chronic ATLL and the utility of both PVL and clonal analysis as a test of the efficacy of novel treatment regimes.

